# Preliminary exploration of metabolomics mechanisms in patients with patent foramen ovale and migraine

**DOI:** 10.22514/jofph.2024.044

**Published:** 2024-12-12

**Authors:** Parhati Tuerxun, Juanli Liu, Adilai Aisaiti, Guzhuo Shen, Pengfei Gong, Xiufen Li

**Affiliations:** ^1^Department of Cardiology, Xinjiang Medical University Affiliated Traditional Chinese Medicine Hospital, 830001 Urumqi, Xinjiang Uyghur Autonomous Region, China; ^2^Department of Cardiology, Xinjiang Uyghur Autonomous Region Uyghur Medical Hospital, 830001 Urumqi, Xinjiang Uyghur Autonomous Region, China; ^3^The Fourth Clinical Medical College of Xinjiang Medical University, 830001 Urumqi, Xinjiang Uyghur Autonomous Region, China

**Keywords:** Patent foramen ovale, Migraine; Interventional occlusion, Metabolomics

## Abstract

This study aimed to investigate the metabolic mechanisms underlying the combination of patent foramen ovale (PFO) and migraine by assessing metabolite expression before and after interventional occlusion surgery. The study included 11 PFO patients from the Heart Center of Xinjiang Medical University Affiliated Hospital of Traditional Chinese Medicine, who underwent transcatheter PFO intervention and occlusion surgery between January 2018 and February 2023, and 11 healthy controls. Blood samples were collected pre-surgery, 3 days post-surgery, and 30 days post-surgery for metabolomics analysis. The goal was to identify differentially expressed metabolites between groups. Statistical analyses were performed to evaluate these metabolites alongside migraine disability, assessed using the Migraine Disability Assessment (MIDAS) score. Preliminary analysis of metabolic pathways was also conducted. Results showed significant differences in serum metabolites, including dopamine, L-proline, L-tyrosine, D-proline, acetylcarnitine, and dulcitol, between PFO migraine patients and healthy controls based on Liquid Chromatography-Mass Spectrometry (LC-MS) non-targeted metabolomics analysis. Kyoto Encyclopedia of Genes and Genomes (KEGG) pathway analysis of these metabolites revealed enrichment in protein digestion, absorption, and metabolic signaling pathways, highlighting the role of metabolism in the disease process. Elevated levels of dopamine and other metabolites were found in migraine patients, with differential metabolites primarily associated with the arginine metabolic pathway, suggesting its importance in the condition’s progression. Additionally, patients with PFO and migraine showed significant improvements in headache frequency, duration, and severity post-treatment (*p* < 0.05), though accompanying symptoms did not show statistically significant changes (*p* > 0.05). Overall, interventional closure surgery for PFO significantly alleviates headache symptoms in patients.

## 1. Introduction

Patent foramen ovale (PFO) is a common congenital heart defect caused by incomplete closure of the atrial septum during fetal development, affecting approximately 20% to 35% of the general population [1]. In recent years, PFO has been widely studied for its potential association with various medical conditions, particularly migraine, especially migraine with aura. Migraine is a prevalent primary headache disorder known to significantly impact health and quality of life. Research indicates that the prevalence of PFO is significantly higher among migraine patients, especially those with aura, compared to the general population. This finding has sparked considerable interest in the potential pathophysiological connection between PFO and migraine [2].

According to the Global Burden of Disease Study 2019, migraine is one of the leading causes of disability worldwide, affecting millions and significantly diminishing quality of life [3]. Despite extensive research, the efficacy of PFO closure in alleviating migraine symptoms remains controversial. While some studies have shown significant improvement in migraine frequency and severity following PFO closure, others, including randomized controlled trials, have produced inconsistent results. This inconsistency underscores the need for further research to clarify the role of PFO in migraine management [4].

Metabolomics, an emerging technique that systematically analyzes small molecules within biological systems, has shown great promise in uncovering the underlying mechanisms of diseases. Although research on metabolomics in PFO-related migraines is still limited, it offers the potential to identify changes in metabolites before and after PFO closure, providing insight into how these changes might influence migraine development. By leveraging metabolomics, researchers can gain a deeper understanding of the role of PFO in migraine pathogenesis and identify new therapeutic targets.

This study aims to compare the MIDAS (Migraine Disability Assessment) scores of PFO patients before and after interventional closure and analyze the metabolomic profiles of these patients compared to healthy controls. By investigating the differential expression of small molecules, we hope to elucidate the mechanisms underlying PFO-related migraine and provide new insights for clinical management strategies.

## 2. Materials and methods

A total of 11 PFO inpatients with migraine complicated with transcatheter closure of PFO at the Heart Center of the Affiliated Hospital of Traditional Chinese Medicine of Xinjiang Medical University from January 2018 to February 2023 were collected. All of them were treated with transcatheter PFO interventional occlusion as the observation group, and 11 healthy controls matched with general data were selected for metabolomics detection.

The inclusion criteria for the study required that patients exhibit a right-to-left shunt (RLS grade 2–3) as confirmed by transthoracic echocardiography and that PFO be verified during the operation with subsequent intervention. Healthy controls were required to have no PFO, confirmed by echocardiography or contrast echocardiography of the right heart. The exclusion criteria were as follows: pregnant or lactating women, patients with infectious diseases, individuals with severe cerebrovascular conditions, arrhythmia, cardiopulmonary insufficiency, cardiac valvular disease, pulmonary hypertension or other serious health conditions, as well as those with poor compliance or incomplete data, malignant tumors, or headaches due to other underlying diseases.

The study adhered to the ethical guidelines of the Declaration of Helsinki and was approved by the Ethics Committee of the Affiliated Hospital of Traditional Chinese Medicine of Xinjiang Medical University (Approval No. 2023XE-GS150). All participants provided informed consent prior to their inclusion in the study.

The diagnostic criteria were established based on two main assessments. First, contrast echocardiography of the right heart was used to determine the degree of right-to-left shunt. The shunt volume was calculated by evaluating the transient unidirectional high signal on the ultrasound spectrum, with the volume recorded after two Valsalva maneuvers being used to classify the shunt. The classification of the degree of right-to-left shunt was as follows: Level 0 indicated no bubbles, Level 1 indicated 1–10 bubbles, Level 2 indicated 10–30 bubbles and Level 3 indicated more than 30 bubbles. Second, the diagnostic criteria for simple migraine adhered to the “Chinese Guidelines for the Diagnosis and Treatment of Migraine”, as outlined in the first edition by the Neurology Branch of the Chinese Medical Association [5]. Baseline clinical data, including sex, age and body mass index (BMI), were collected for all patients. The surgical procedure involved puncturing the right femoral vein under local anesthesia to introduce a catheter and guide wire into the right atrium, which was then advanced through the foramen ovale to the left atrium. Following a successful operation, patients were administered postoperative medications, including low molecular weight heparin, aspirin and clopidogrel. Blood samples were collected from PFO patients (5 mL each) before the procedure, as well as 3 days and 30 days after surgery. For non-PFO patients, 5 mL of peripheral venous blood was collected directly for metabolomics analysis. Metabolomics was performed using high-performance liquid chromatography-tandem mass spectrometry (LC-MS/MS).

The Migraine Disability Questionnaire (MIDAS score) was used to assess the impact of headaches on daily life for all migraine patients. The migraine score includes five parts: (1) Frequency of headache attacks: 0 points—no attacks, 3 points—≤2 attacks per month, 6 points—3–4 attacks per month, 9 points—≥5 attacks per month (recurrent headaches within 48 hours are considered a single episode); (2) Duration of headache: 0 points—no attacks, 3 points—average monthly duration ≤12 hours, 6 points—average monthly duration ≥12 hours but <2 days, 9 points—average monthly duration >2 days; (3) Severity of headache: 0 points—no pain, 3 points (mild), 6 points (moderate), 9 points (severe); (4) Accompanying symptoms such as nausea, vomiting, photophobia, and phonophobia: 1 point for each symptom, up to a maximum of 3 points; (5) Other symptoms: 0 points—none, 1 point—present. Evaluations were conducted before and 30 days after PFO occlusion. Serum samples were pretreated and analyzed using High-Performance Liquid Chromatography-Mass Spectrometry/Mass Spectrometry (HPLC-MS/MS). Data processing was carried out with ProteoWizard and XCMS programs, and statistical analyses were performed using SPSS 24.0 (IBM Corporation, Armonk, NY, USA) software. Principal Component Analysis (PCA), Partial Least Squares Discriminant Analysis (PLS-DA), and Orthogonal Partial Least Squares Discriminant Analysis (OPLS-DA) were employed for pattern recognition and data interpretation.

## 3. Results

### 3.1 Baseline data

The study comprised 11 patients with PFO undergoing transcatheter PFO closure and 11 healthy controls, making a total of 22 participants. Data analysis showed no statistically significant differences between the two groups in terms of gender, age and BMI (*p* > 0.05) (Table 1).

**Table 1.** Baseline characteristic comparison between the non-PFO and PFO group.

Variables (N = Total patient population)	Non-PFO (N = 11)	PFO (N = 11)	*p*
Sex (male)	5 (45.4)	4 (36.3)	0.97
Age (<55 years old)	6 (54.5)	5 (45.5)	0.67
BMI (kg/m^2^)	22.6 ± 2.2	22.7 ± 2.5	0.93

*PFO: patent foramen ovale; BMI: body mass index.*

### 3.2 Changes in MIDAS before and after PFO occlusion surgery

Data analysis further revealed that the frequency, duration, and severity of headaches in patients with PFO complicated by migraine showed significant improvement post-surgery compared to pre-surgery levels (*p* < 0.05) (Table 2). However, the changes in scores for accompanying symptoms and other symptoms were not statistically significant (*p* > 0.05).

**Table 2.** Pre- and post-operative MIDAS scores of migraine patients with and without PFO.

Score (points)	PFO-pre (N = 11)	PFO-post (N = 11)	*Z*/*t*	*p*
Frequency	9.093 (0, 9.0)	3.090 (0, 3.0)	−2.123	0.032
Time	6.093 (0, 9.0)	3.090 (0, 3.0)	−2.086	0.044
Degree	6.093 (0, 6.0)	3.090 (0, 3.0)	−1.983	0.047
Concomitant symptom	1.090 (0, 1.0)	0.090 (0, 1.0)	−1.414	0.157
Other symptoms	0.090 (0, 0.0)	0.090 (0, 1.0)	−1.732	0.083
Total points	16.9 ± 9.8	8.6 ± 6.2	2.246	0.048

*PFO: patent foramen ovale.*

### 3.3 UPLC-QTOF-MS conditional verification

The basic peak intensity ion flow graphs were compared and overlapped to assess consistency. The results showed that the total ion chromatograms (TIC) for all samples were generally similar, with the response intensities and retention times of each chromatographic peak demonstrating substantial overlap, indicating that the variation introduced by instrument errors during the experiment was minimal (Fig. 1).

**Fig. 1.** TIC spectra for positive and negative ion modes. The abscissa represents the retention time, and the ordinate represents the relative strength of the ion. TIC: total ion chromatograms.

### 3.4 Establishment of metabolic profile discrimination model

The serum samples from 11 negative controls and 11 patients with PFO were analyzed using LC-MS non-targeted metabolomics, and the samples included those taken before, 3 days after and 30 days after the operation. The data were subjected to modeling and discriminant analysis using both unsupervised and supervised methods. In addition, PCA and Orthogonal OPLS-DA were employed to establish the discriminant model.

#### 3.4.1 Establishment of the PCA model

The PCA plot (Fig. 2) shows a distinct trend of separation between samples under positive and negative ion modes. Specifically, the second principal component (t [2]) illustrates that most negative controls are clustered on the far left, while the PFO patient samples are predominantly positioned on the right. This separation indicates a significant difference in metabolic states between PFO patients and the control group. Moreover, notable separation and clustering of PFO patient samples were observed across the different time points (before the operation, 3 days after the operation, and 30 days after the operation), suggesting distinct serum metabolic profiles at each stage of the postoperative period.

**Fig. 2.** PCA score chart for positive and negative ion modes. PCA: Principal Component Analysis.

#### 3.4.2 PLS-DA

The PLS-DA model established for this study, based on both positive and negative ion mode data, demonstrated strong performance. As shown in Table 3, the explanation rate (*R*^2^) and prediction rate (*Q*^2^) for the model were both ≥0.5, indicating that the model is stable and reliable. Fig. 3 illustrates that the PLS-DA model effectively separates the groups, suggesting that the metabolites identified could serve as potential markers for differential diagnosis.

**Table 3.** PLS-DA model stability evaluation parameters.

Model	*R* ^2^	Q^2^
Negative control *vs.* Before operation	0.993	0.930
Before operation *vs.* 3 days after surgery	0.955	0.158
Before operation *vs.* 30 days after surgery	0.933	0.657
3 days after surgery *vs.* 30 days after surgery	0.994	0.565

**Fig. 3.** PLS-DA score chart for positive and negative ion modes. PLS-DA: Partial Least Squares Discriminant Analysis.

#### 3.4.3 OPLS-DA

Fig. 4 illustrates the enhanced separation between sample groups achieved through OPLS-DA. The analysis indicates a clearer distinction between groups compared to previous methods, suggesting that OPLS-DA is effective in differentiating between the sample types. The OPLS-DA model, based on positive and negative ion mode data, shows stability and reliability, as evidenced by the *R*^2^ and *Q*^2^ values presented in Table 4.

**Fig. 4.** OPLS-DA score chart for positive and negative ion modes. OPLS-DA: Orthogonal Partial Least Squares Discriminant Analysis.

**Table 4.** OPLS-DA model stability evaluation parameters.

Model	*R* ^2^	Q^2^
Negative control *vs.* Before operation	0.993	0.932
Before operation *vs.* 3 days after surgery	0.955	0.129
Before operation *vs.* 30 days after surgery	0.999	0.467
3 days after surgery *vs.* 30 days after surgery	0.994	0.182

Fig. 5 illustrates the permutation test plot for the OPLS-DA model, demonstrating that the model established in this experiment has not been overfitted.

**Fig. 5.** Permutation test plot for OPLS-DA model. OPLS-DA: Orthogonal Partial Least Squares Discriminant Analysis.

### 3.5 Screening of differential metabolites

Differential metabolites were identified through multiple analyses of variance and *T*-tests, applying the criteria of fold change (FC) >2.0 and *p* < 0.05. We identified thousands of differential metabolites among the groups, as detailed in Table 5. These include dopamine, L-proline, L-tyrosine, D-proline, acetylcarnitine and dulcitol, which exhibited significant changes among the groups. The results are visualized in a volcano plot, as shown in Fig. 6. The impact and explanatory power of metabolite expression patterns on sample classification were further evaluated using VIP (Variable Importance in Projection) scores derived from the OPLS-DA model. Metabolites were initially screened based on *p* < 0.05 and a VIP score >1 to identify significant differences among the groups. Notable metabolites, including those mentioned above, were highlighted as having significant differential expression, further emphasizing their potential role in the underlying metabolic mechanisms (Table 5).

**Fig. 6.** Volcano plot of differentially expressed metabolites.

**Table 5.** Comparison of significantly different metabolite statistics between different groups.

Model	Total	UP	Down
Negative control *vs.* Before operation	42	39	3
Before operation *vs.* 3 days after surgery	22	4	18
Before operation *vs.* 30 days after surgery	37	13	24
3 days after surgery *vs.* 30 days after surgery	15	8	7

### 3.6 Bioinformatics analysis of differential metabolites

#### 3.6.1 Cluster analysis of differential metabolites

Hierarchical cluster analysis was employed to accurately identify marker metabolites across different samples. With color intensity representing metabolite levels—blue indicates lower content, while red signifies higher content. The heat map distinctly shows that the metabolic profiles of the positive group differ significantly from those of the negative control group, highlighting differences in their metabolic spectra (Fig. 7).

**Fig. 7.** Hierarchical clustering results of differential metabolites.

#### 3.6.2 KEGG pathway analysis for differential metabolites

The observed metabolic differences indicate that various metabolic pathways are disrupted in patients with PFO. To further elucidate these disruptions, we performed enrichment and pathway analysis on the differential metabolites identified in this study, which involved submitting the metabolites to the KEGG database for pathway analysis. Fig. 8 presents the pathways that exhibit the most significant differences in metabolite levels. These pathways include protein digestion and absorption signaling pathways, metabolic signaling pathways, arginine and proline metabolism pathways, ATP (Adenosine Triphosphate)-binding cassette transporter signaling pathways, as well as fatty acid and purine metabolism pathways.

**Fig. 8.** KEGG pathway analysis of significantly differentiated metabolites. The figure displays the metabolic pathways with the most notable differences across various conditions. The upper left panel compares the negative control group to the pre-operative samples. The upper right panel shows comparisons between pre-operative samples and those collected three days post-surgery. The lower left panel illustrates differences between pre-operative samples and those collected thirty days post-surgery. The lower right panel contrasts samples collected three days post-surgery with those collected thirty days post-surgery. Each pathway is color-coded to represent the level of significance in the differences observed. tRNA: Transfer Ribonucleic Acid; ABC: Adenosine Triphosphate-Binding Cassette.

#### 3.6.3 Metabolites with biomarker significance

Significant differential metabolites were identified from serum samples across the various groups, including the negative control group versus migraine patients with PFO before the operation, migraine patients with PFO before and 3 days after surgery, and migraine patients with PFO 30 days after versus before surgery. Nonparametric tests were employed to evaluate the levels of these metabolites across the groups. On this basis, metabolites exhibiting differences across all groups were identified, including dopamine, L-proline, L-tyrosine, D-proline, acetylcarnitine and dulcitol. These six metabolites showed significant differential expression among the groups. The results are presented in a box plot (Fig. 9), where the x-axis represents the subject groups and the y-axis indicates metabolite levels. The plot demonstrates that serum levels of D-proline, glycerol ester, and xanthine were lower in the negative control group compared to the PFO group, with further reduction observed in samples taken 3 and 30 days after surgery. Conversely, serum galactosyl levels were higher in the negative control group than in the PFO group and increased further in patients with PFO after surgery compared to pre-operative levels.

**Fig. 9.** Representative biomarkers of differential metabolites. *: *p* < 0.05. **: *p* < 0.01. ***: *p* < 0.001. ****: *p* < 0.0001.

## 4. Discussion

This study utilized metabolomics to analyze the differences in metabolites between PFO-associated migraine patients and healthy controls. Significant differences were found in metabolites such as dopamine, L-proline, L-tyrosine, D-proline and acetylcarnitine. KEGG pathway analysis indicated that these differential metabolites were enriched in pathways related to protein digestion and absorption, metabolic signaling, ATP-binding cassette transporters, arginine and proline metabolism, and other related pathways.

Recent studies on migraine metabolomics are consistent with our findings [5, 6]. For example, Aczél T *et al*. [7] reported that certain metabolites, such as lactate, exhibit significant differences in migraine patients, likely due to disruptions in glucose metabolism and energy metabolism. Our study further confirms that many of these differential metabolites are enriched in energy metabolism pathways. However, our results suggest that dopamine and arginine metabolism pathways play a critical role in the progression of migraines in PFO patients, which differs from some existing reports.

Additionally, there are already studies that demonstrate a direct relationship between PFO and migraines. Liu *et al*. [8] found that certain brain regions in PFO patients exhibited significant alterations in functional connectivity, suggesting that PFO might trigger migraines by altering brain metabolic activity. Lei *et al*. [9] discovered that PFO might influence cortical excitability in migraine patients, especially in the occipital lobe, with PFO-associated migraine patients showing increased EEG band power under visual stimulation, further supporting the pathophysiological role of PFO in migraines.

Our study also showed that PFO closure surgery significantly improved headache frequency, duration and severity, although accompanying symptoms, such as nausea and photophobia, did not show statistically significant improvement. Dopamine, a key neurotransmitter involved in regulating various brain functions such as mood, motor control, and pain perception, may play a critical role in the occurrence and aggravation of headaches in migraine patients due to metabolic disturbances [10, 11]. In our study, dopamine levels were significantly elevated in PFO-associated migraine patients, suggesting that dopamine metabolism plays a crucial role in migraine attacks. KEGG pathway analysis also indicated that dopamine metabolism is closely related to protein digestion, absorption, and signaling pathways, which may reflect broader metabolic changes in migraine patients.

Additionally, arginine, as a precursor of nitric oxide (NO), plays a critical role in vasodilation [12]. Abnormal arginine metabolism, particularly increased NO production, may lead to cerebral blood flow dysregulation in migraine patients, triggering headache attacks. KEGG analysis indicated significant changes in the arginine metabolic pathway in PFO migraine patients, suggesting that this pathway is an important mechanism in migraine pathophysiology [13, 14].

L-proline and D-proline are involved in protein synthesis and cellular metabolism, particularly in the nervous system [15]. Our study found significant changes in L-proline and D-proline levels in PFO-associated migraine patients, suggesting that proline metabolism may be related to neurotransmitter regulation and neuroinflammation, further exacerbating migraine attacks. Acetylcarnitine plays a key role in transporting fatty acids and generating energy in the mitochondria [16]. Elevated acetylcarnitine levels observed in PFO-associated migraine patients suggest potential energy metabolism impairment, especially during migraine attacks, leading to insufficient energy supply and worsening headache symptoms.

KEGG pathway analysis also showed that protein digestion and absorption pathways were enriched among the differential metabolites, indicating that systemic metabolism in migraine patients may be widely affected. Disruptions in protein metabolism can affect neurotransmitter production and cellular function, further destabilizing the nervous system and worsening migraine symptoms [17, 18].

Migraine not only significantly affects the quality of life but is also associated with severe complications such as white matter lesions, cognitive decline, and cerebral infarction. Understanding the pathogenesis of migraine and developing individualized treatments has important clinical and societal value. PFO affects approximately 20%–34% of adults and is closely associated with migraines, although the underlying mechanisms remain unclear [6]. Several hypotheses have been proposed to explain the connection between PFO and migraines, including microembolism, cerebral blood flow dysregulation and genetic factors [19]. The “2022 Chinese Consensus on Echocardiographic Diagnosis of Patent Foramen Ovale” emphasizes the key role of PFO closure in the treatment of migraines [20]. The results of this study are consistent with recent PRIMA and PREMIUM studies, supporting the efficacy of PFO closure in alleviating migraine symptoms. However, the 2022 Consensus also pointed out that migraines are multifactorial, and PFO closure alone may not be a comprehensive solution. It is necessary to consider the complexity of migraine pathogenesis when formulating treatment plans.

This study has several limitations. First, the small sample size may limit the generalizability of the findings. Second, the study did not fully capture the dynamic changes in various metabolites within metabolic pathways. Future research should aim to increase the sample size, further explore the key mechanisms in PFO-associated migraines and identify specific metabolic targets for treatment.

## 5. Conclusions

This study sheds light on the metabolic mechanisms underlying the combination of patent foramen ovale (PFO) and migraine by exploring differential metabolites before and after PFO occlusion surgery. The findings indicate that PFO closure significantly improves migraine symptoms, particularly in terms of headache frequency, duration, and severity, as measured by the MIDAS score. Notable metabolic differences were identified between PFO migraine patients and healthy controls, with key metabolites such as dopamine, L-proline, and acetylcarnitine showing significant variation. These results suggest that metabolic pathways, particularly those related to arginine and proline metabolism, may play a crucial role in migraine pathogenesis in PFO patients. While this study provides important insights, further research with larger sample sizes is needed to confirm these findings and explore specific metabolic targets for therapeutic interventions in PFO-related migraine.

## References

[1] Tang Y, Sun H, Plummer C, Vogrin SJ, Li H, Li Y, *et al*. Association between patent foramen ovale and migraine: evidence from a resting-state fMRI study. Brain Imaging and Behavior. 2024; 18: 720–729. 10.1007/s11682-024-00868-9 PMC11364569 38381323

[2] Beneki E, Dimitriadis K, Aznaouridis K, Soulaidopoulos S, Kostakis P, Sakalidis A, *et al*. Patent foramen ovale closure reduces migraine burden: a meta-analysis. European Heart Journal. 2023; 44: ehad655.2269.

[3] Tobis JM, Charles A, Silberstein SD, Sorensen S, Maini B, Horwitz PA, *et al*. Percutaneous closure of patent foramen ovale in patients with migraine: the PREMIUM trial. Journal of the American College of Cardiology. 2017; 70: 2766–2774. 10.1016/j.jacc.2017.09.1105 29191325

[4] Mattle HP, Evers S, Hildick-Smith D, Becker WJ, Baumgartner H, Chataway J, *et al*. Percutaneous closure of patent foramen ovale in migraine with aura, a randomized controlled trial. European Heart Journal. 2016; 37: 2029–2036. 10.1093/eurheartj/ehw027 26908949

[5] Chinese Society of Neurology, Chinese Society of Neurology Headache Collaboration Group. Chinese guidelines for the diagnosis and treatment of migraine (Chinese society of neurology, first edition). Chinese Journal of Neurology. 2023; 56: 591–613.

[6] Zhang YS, Yu SY, Dong Z, He L, Zhu H, Bai Y. Chinese expert consensus on the prevention and treatment of patent foramen oval related non-apoplectic disease. Chinese Heart Journal. 2024; 36: 125–134.

[7] Aczél T, Körtési T, Kun J, Urbán P, Bauer W, Herczeg R, *et al*. Identification of disease-and headache-specific mediators and pathways in migraine using blood transcriptomic and metabolomic analysis. The Journal of Headache and Pain. 2021; 22: 117. 10.1186/s10194-021-01285-9 PMC8493693 34615455

[8] Lei X, Wei M, Qi Y, Wang L, Liu C, Guo Y, *et al*. The patent foramen ovale may alter migraine brain activity: a pilot study of electroencephalography spectrum and functional connectivity analysis. Frontiers in Molecular Neuroscience. 2023; 16: 1133303. 10.3389/fnmol.2023.1133303 PMC10029922 36959871

[9] Cao W, Jiao L, Zhou H, Zhong J, Wang N, Yang J. Right-to-left shunt-associated brain functional changes in migraine: evidences from a resting-state FMRI study. Frontiers in Human Neuroscience. 2024; 18: 1432525. 10.3389/fnhum.2024.1432525 PMC11392749 39281370

[10] Yan C, Li H, Wang C, Yu H, Guo T, Wan L, *et al*. Frequency and size of* in situ* thrombus within patent foramen ovale. Stroke. 2023; 54: 1205–1213. 10.1161/STROKEAHA.122.041524 36891906

[11] Kheiri B, Abdalla A, Osman M, Ahmed S, Hassan M, Bachuwa G, *et al*. Percutaneous closure of patent foramen ovale in migraine: a meta-analysis of randomized clinical trials. JACC: Cardiovascular Interventions. 2018; 11: 816–818. 10.1016/j.jcin.2018.01.232 29673517

[12] Trabattoni D, Brambilla M, Canzano P, Becchetti A, Teruzzi G, Porro B, *et al*. Migraine in patients undergoing PFO closure: characterization of a platelet-associated pathophysiological mechanism: the LEARNER study. JACC: Basic to Translational Science. 2022; 7: 525–540. 10.1016/j.jacbts.2022.02.002 PMC9270571 35818509

[13] Wintzer-Wehekind J, Horlick E, Ibrahim R, Cheema AN, Labinaz M, Nadeem N, *et al*. Effect of clopidogrel and aspirin *vs.* aspirin alone on migraine headaches after transcatheter atrial septal defect closure: one-year results of the CANOA randomized clinical trial. JAMA Cardiology. 2021; 6: 209–213. 10.1001/jamacardio.2020.4297 PMC7512130 32965476

[14] Wu T, Zhang KY, Yao JY, Li YJ. Comparative study of right heart echocardiography and transcranial Doppler bubble test in diagnosing patent foramen ovale in patients with cryptogenic stroke. Journal of Ultrasound in Clinical Medicine. 2023; 25: 395–399.

[15] Mojadidi MK, Kumar P, Mahmoud AN, Elgendy IY, Shapiro H, West B, *et al*. Pooled analysis of PFO occluder device trials in patients with PFO and migraine. Journal of the American College of Cardiology. 2021; 77: 667–676. 10.1016/j.jacc.2020.11.068 33573735

[16] Antonova M. Prostaglandins and prostaglandin receptor antagonism in migraine. Danish Medical Journal. 2013; 60: B4635. 10.1186/1129-2377-14-s1-p114 23673269

[17] Barbanti P, Aurilia C, Egeo G, Fofi L, Guadagni F, Ferroni P. Dopaminergic symptoms in migraine: a cross-sectional study on 1148 consecutive headache center-based patients. Cephalalgia. 2020; 40: 1168–1176. 10.1177/0333102420929023 32484361

[18] Ferroni P, Zanzotto FM, Scarpato N, Spila A, Fofi L, Egeo G, *et al*. Machine learning approach to predict medication overuse in migraine patients. Computational and Structural Biotechnology Journal. 2020; 18: 1487–1496. 10.1016/j.csbj.2020.06.006 PMC7327028 32637046

[19] D’Andrea G, Gucciardi A, Perini F, Leon A. Pathogenesis of cluster headache: from episodic to chronic form, the role of neurotransmitters and neuromodulators. Headache. 2019; 59: 1665–1670. 10.1111/head.13673 31603552

[20] Chinese Expert Consensus Group on The Clinical Application of Transesophageal Echocardiography. Chinese expert consensus on right heart echocardiography with patent foramen ovale. China Circulation Journal. 2022; 37: 449–458.

